# Surgical Risk and Long-Term Mortality With PCI and CABG in Ischemic Left Ventricular Systolic Dysfunction

**DOI:** 10.1016/j.jscai.2025.103820

**Published:** 2025-08-19

**Authors:** Guillaume Marquis-Gravel, Guangyu Tong, Matthew Dodd, Tim Clayton, Matthew Ryan, Kieran F. Docherty, Alicia Williams, Jiaxuan Sun, Stephen E. Fremes, Alexandra J. Lansky, Eric J. Velazquez, Divaka Perera, Mark C. Petrie, Jean-Lucien Rouleau

**Affiliations:** a Department of Medicine, Montreal Heart Institute, Université de Montréal, Montreal, Canada; b Yale School of Medicine, New Haven, Connecticut; c Department of Medical Statistics, London School of Hygiene and Tropical Medicine, London, United Kingdom; d Kings College London, British Heart Foundation Centre of Research Excellence, St Thomas' Hospital, and Cardiovascular Division, Guy's and St Thomas' NHS Foundation Trust, London, United Kingdom; e School of Cardiovascular and Metabollic Health, University of Glasgow, Glasgow, Scotland, United Kingdom; f Yale School of Public Health, New Haven, Connecticut; g Department of Surgery, Sunnybrook Health Sciences Centre, University of Toronto, Toronto, Canada

**Keywords:** coronary artery bypass graft, coronary artery disease, heart failure, percutaneous coronary intervention, revascularization

## Abstract

**Background:**

Coronary artery bypass grafting (CABG) improves survival compared with optimal medical therapy (OMT) alone in patients with ischemic left ventricular systolic dysfunction (iLVSD), but percutaneous coronary intervention (PCI) did not show clinical benefits in this population. However, the randomized controlled trials (RCT) evaluating these 2 revascularization modalities may differ in terms of baseline surgical risk. The aim is to investigate whether the treatment effects of PCI vs OMT, and of CABG vs OMT, are modified by baseline surgical risk.

**Methods:**

A post hoc analysis of the Revascularization for Ischemic Ventricular Dysfunction – British Cardiovascular Intervention Society 2 (REVIVED-BCIS2) and Surgical Treatment for Ischemic Heart Failure (STICH) RCT comparing PCI and CABG vs OMT, respectively, in patients with iLVSD, was conducted. The main outcome was all-cause mortality. Interaction between randomized treatment and baseline surgical risk, estimated by a modified European System for Cardiac Operative Risk Evaluation (EuroSCORE)-II, was quantified.

**Results:**

A total of 666 participants from the REVIVED-BCIS2 trial and 1200 participants from the STICH trial were included. Participants from the REVIVED-BCIS2 trial were more likely to be in the highest tertile of baseline EuroSCORE-II (40.4% vs 29.4%, respectively; *P* < .001). In the REVIVED-BCIS2 trial, PCI had a consistent lack of effect on all-cause mortality vs OMT across baseline EuroSCORE-II tertiles (*P* for interaction = .79). In the STICH trial, CABG reduced mortality consistently vs OMT across baseline EuroSCORE-II tertiles (*P* for interaction = .64).

**Conclusions:**

In the 2 largest RCT evaluating the impact of revascularization in iLVSD and multivessel coronary disease, the treatment effect of PCI vs OMT, and of CABG vs OMT, was not modified by baseline surgical risk.

## Introduction

Coronary artery bypass grafting (CABG) is recommended by international clinical practice guidelines as the first-line revascularization modality in most patients with heart failure (HF) and chronic ischemic left ventricular (LV) systolic dysfunction,^1^, ^2^, ^3^, ^4^ whereas percutaneous coronary intervention (PCI) is suggested as a reasonable alternative for symptomatic patients for whom surgery is not considered appropriate.^1^^,^^3^ These recommendations are based on the Surgical Treatment for Ischemic Heart Failure (STICH) trial, which showed that CABG reduced all-cause mortality, cardiovascular mortality, and hospitalization for cardiovascular causes compared with optimal medical therapy (OMT) alone over long-term follow-up.^5^^,^^6^ Although STICH participants were deemed to be suitable surgical candidates, 5.1% of those who underwent CABG died within 30 days of the surgery.^7^ It is thus possible that CABG may have differential treatment effects in patients with varying baseline surgical risk. Patients with lower operative risk might be more likely to survive the index procedure and gain in terms of reduced long-term mortality, whereas patients with high upfront operative risk may fare worse with CABG.

In recent decades, revascularization techniques with PCI have iteratively improved with the development of novel stent platforms, physiology-guided PCI, intravascular imaging, transradial approach, complete total occlusion techniques, atherectomy devices, and safer antiplatelet strategies.^8^^,^^9^ Given these advances, PCI is frequently used to treat patients with ischemic LV systolic dysfunction in routine clinical practice,^10^, ^11^, ^12^ despite guidelines recommendations preferentially recommending CABG.^1^, ^2^, ^3^, ^4^ However, the recent Revascularization for Ischemic Ventricular Dysfunction – British Cardiovascular Intervention Society 2 (REVIVED-BCIS2) trial suggested that PCI did not reduce the composite of all-cause death or HF hospitalizations (or the individual components of this primary end point) compared with OMT alone in this population.^13^ Whether patients enrolled in the REVIVED-BCIS-2 and STICH trials, the only 2 randomized controlled trials (RCT) evaluating revascularization strategies in patients with ischemic LV systolic dysfunction, differed in terms of baseline risk is unknown. Our objective was to evaluate whether the treatment effect on all-cause mortality of PCI vs OMT, and of CABG vs OMT, differed across the spectrum of baseline surgical risk in patients with HF, LV systolic dysfunction, and multivessel disease.

## Materials and methods

### Study design

A post hoc analysis of the REVIVED-BCIS2 (ClinicalTrials.gov; NCT01920048) and STICH (ClinicalTrials.gov; NCT00023595) trials was performed. REVIVED-BCIS2 was a multicenter, open-label RCT conducted in the United Kingdom comparing contemporary PCI vs OMT alone in 700 patients with an ejection fraction ≤35%, extensive coronary disease, and who demonstrated myocardial viability in the segments to be revascularized from 2013-2020, with a median follow-up of 41 months.^13^ STICH was an international, multicenter, open-label RCT comparing CABG vs OMT alone in 1212 patients with an ejection fraction ≤35% and coronary artery disease suitable for CABG from 2002-2007.^5^ For this analysis, we report the long-term follow-up extension (median of 9.8 years) of the STICH trial, the STICH Extended study (STICHES).^6^

Baseline surgical risk was estimated using a modified European System for Cardiac Operative Risk Evaluation (EuroSCORE)-II. Some variables of the EuroSCORE-II required approximations to accommodate the data available in each study data set (Supplemental Table S1). No approximation was required for most variables (age, sex, creatinine clearance, diabetes on insulin, CCS class 4 angina, LV ejection fraction, New York Heart Association class, surgery on thoracic aorta, dialysis [in REVIVED-BCIS2], and weight of operation [in REVIVED-BCIS2]). Chronic lung disease, active endocarditis, and dialysis (in STICH) were not captured within the data sets and were all imputed as absent. The main outcome of this analysis was all-cause mortality (STICH trial primary end point). The secondary outcomes were cardiovascular death, hospitalization for HF, and the composite of all-cause death or HF hospitalization (REVIVED-BCIS2 primary end point). The definitions of the clinical outcomes in both trials are provided in Supplemental Table S2.

### Statistical analysis

The primary analyses were performed in the intention-to-treat cohorts. Baseline EuroSCORE-II tertiles (T1, T2, and T3) were calculated from the pooled data sets of both trials to ensure that the tertile boundaries were the same in both trials. The analyses of outcomes were performed separately in the REVIVED-BCIS2 and STICH cohorts to allow comparability of the treatment group with its own control group. Baseline variables were compared between trials using a χ^2^ test for categorical variables, a 2-sample *t* test for continuous variables, and a Wilcoxon rank-sum test for EuroSCORE-II due to the skewness of the data. Cox proportional hazards analysis was conducted to evaluate the treatment effect of revascularization (PCI or CABG) based on baseline EuroSCORE-II, after confirming that the proportional hazards assumptions were met by comparison of –log(-log) estimated survivor curves. Hazard ratios (HR) for comparison of PCI or CABG vs OMT and 95% confidence intervals (CI) were calculated in the overall cohort, and separately in the 3 tertiles of EuroSCORE-II. Competing risk of death was accounted for using Fine-Gray modeling for hospitalization and CV death outcomes. The interaction term was included in the model, and the *P* value for interaction was calculated to evaluate the potential treatment effect modification with EuroSCORE-II modeled as a continuous variable and by tertiles. Cumulative incidence graphs of all-cause mortality (STICH primary end point) and of the composite of all-cause mortality or HF hospitalization (REVIVED-BCIS2 primary end point) were generated for each surgical risk tertile. Cumulative incidence graphs by tertiles with a landmark at 30 days after randomization were also generated. A sensitivity analysis, imputing different values to missing EuroSCORE-II variables, was conducted to evaluate the impact of missingness in baseline variables on the findings of the primary analysis. Multiple imputation was performed 10 times for missing entries using baseline covariates, intervention, and outcomes, for each trial separately. EuroSCORE-II tertiles were established based on the imputed EuroSCORE-II for each data set. Cox regression models were fitted to each of the 10 imputed data sets for each trial. Pooled HR and 95% CI were obtained using Rubin's rules.^14^ For the interaction test, the median *P* values across the 10 imputations were reported.^15^ A restricted mean survival time analysis was also conducted by EuroSCORE-II tertiles. This method is useful for scenarios where an intervention is associated with an early effect on the outcomes, as may be observed for early postoperative/procedural mortality after CABG or PCI vs OMT alone. Statistical analyses were performed using R 4.3.1, with no adjustment for multiple comparisons in this exploratory analysis. The research was carried out in accordance with the appropriate ethical guidelines. Both trials were approved by a research ethics committee, and participants provided their informed consent.

## Results

### Patient characteristics

A total of 666 of 700 (95.1%) participants from the REVIVED-BCIS2 trial and 1200 of 1212 (99.0%) participants from the STICH trial had no missing values to calculate the EuroSCORE-II and were included in the analysis. Participants in the REVIVED-BCIS2 trial were older (69.4 ± 9.1 vs 60.3 ± 9.3 years, respectively; *P* < .001) compared with participants from the STICH trial (Table 1). Median EuroSCORE-II in the pooled data set was 2.2% (T1: 1.2%; T2: 2.2%; T3: 4.4%). Baseline EuroSCORE-II was higher in the REVIVED-BCIS2 trial compared to the STICH trial, with a higher proportion of participants in the highest EUROSCORE-II tertile (40.4% vs 29.4%, respectively; *P* < .001). Median follow-up was 3.4 years in the REVIVED-BCIS2 trial and 6.8 years in the STICH trial.

**Table 1:** Baseline characteristics for the REVIVED-BCIS2 and STICH trials.^a^

	REVIVED-BCIS2 (n = 666)	STICH (n = 1200)	*P* value
Age, y	69.4 ± 9.1	60.3 ± 9.3	<.001
Sex			.804
Female	85 (12.8)	147 (12.2)	
Male	581 (87.2)	1053 (87.8)	
LVEF, %	27.0 ± 6.7	26.5 ± 6.0	.14
Baseline EuroSCORE-II surgical risk	2.5% (1.7%-4.1%)	2.0% (1.3%-3.2%)	<.001
EuroSCORE-II tertile			<.001
Low (score <1.2%)	162 (24.3)	459 (38.2)	
Mid (score 1.2%-4.2%)	235 (35.3)	388 (32.3)	
High (score >4.2%)	269 (40.4)	353 (29.4)	
Body mass index, kg/m^2^	28.5 ± 5.4	27.3 ± 4.8	<.001
Hypertension	370 (55.6)	722 (60.2)	.059
Diabetes	270 (40.5)	472 (39.3)	.645
Previous MI	353 (53.0)	924 (77.0)	<.001
Prior CABG	33 (5.0)	36 (3.0)	.044
NYHA			<.001
I	124 (18.6)	136 (11.3)	
II	367 (55.1)	621 (51.8)	
III	165 (24.8)	408 (34.0)	
IV	10 (1.5)	35 (2.9)	
No. of diseased vessels^b^			<.001
1	66 (9.9)	280 (23.4)	
2	330 (49.5)	459 (38.3)	
3	268 (40.2)	435 (36.3)	
Heart failure therapy at baseline			
Beta-blocker	603 (90.5)	1027 (85.6)	.003
ACE inhibitor or angiotensin receptor blocker	552 (82.9)	1074 (89.5)	<.001
Diuretic (potassium sparing)	346 (49.6)	551 (45.9)	.12
ICD	137 (20.6)	29 (2.4)	<.001
SGLT2i	0 (0)	0 (0)	—

Values are mean ± SD, median (IQR), or n (%).

ACE, angiotensin-converting enzyme; CABG, coronary artery bypass grafting; EuroSCORE, European System for Cardiac Operative Risk Evaluation; ICD, implantable cardioverter-defibrillator; LVEF, left ventricular ejection fraction; MI, myocardial infarction; NYHA, New York Heart Association; REVIVED-BCIS2, Revascularization for Ischemic Ventricular Dysfunction – British Cardiovascular Intervention Society 2; SGLT2i, sodium-glucose cotransporter 2 inhibitor; STICH, Surgical Treatment for Ischemic Heart Failure.

a Due to missing data for components of the EuroSCORE-II, the sample sizes differ from the original trials. Poor mobility and creatinine clearance were the 2 variables most frequently missing.

b Represents the number of diseased vessels with stenosis ≥75% in the STICH trial, and ≥70% in the REVIVED-BCIS2 trial.

**Figure 1:**
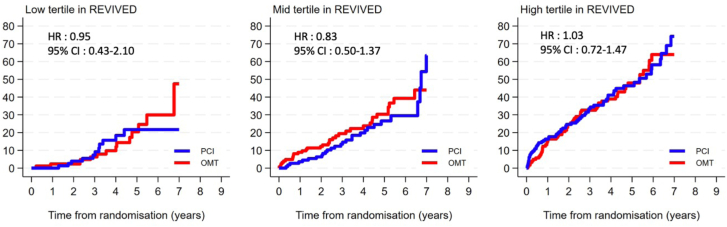
**All-cause mortality by tertiles of European System for Cardiac Operative Risk Evaluation (EuroSCORE)-II with percutaneous coronary intervention (PCI) or optimal medical therapy (OMT) in the Revascularization for Ischemic Ventricular Dysfunction – British Cardiovascular Intervention Society 2 (REVIVED-BCIS2) trial.** PCI did not reduce all-cause mortality consistently across tertiles of baseline EuroSCORE-II (*P* for interaction = .793). HR, hazard ratio.

In the REVIVED-BCIS2 trial, 210 (31.5%) participants experienced the main outcome of all-cause mortality, including 105 (31.4%) in the PCI group and 105 (31.6%) in the OMT group (HR, 1.00; 95% CI, 0.76-1.31; *P* = .993). The treatment effect of PCI vs OMT alone was consistent across baseline EuroSCORE-II values analyzed both as a continuous variable (*P* for interaction = .140) and by tertiles (T1: HR, 0.95 [95% CI, 0.43-2.10]; T2: HR, 0.83 [95% CI, 0.50-1.37]; T3: HR, 1.03 [95% CI, 0.72-1.47]; *P* for interaction = .793) (Table 2 and Central Illustration). Cumulative incidence plots of all-cause mortality by PCI vs OMT are shown by EuroSCORE-II tertiles in Figure 1. In the STICH trial, 730 (60.8%) patients experienced the main outcome of all-cause mortality, including 345 (57.1%) in the CABG group, and 385 (64.6%) in the OMT group (HR, 0.84; 95% CI, 0.72-0.97; *P* = .015). The treatment effect of CABG vs OMT alone was consistent across baseline EuroSCORE-II values analyzed both as a continuous variable (*P* for interaction = .273) and by tertiles (T1: HR, 0.75 [95% CI, 0.57-0.97]; T2: HR, 0.89 [95% CI, 0.69-1.14]; T3: HR, 0.84 [95% CI, 0.66-1.07]; *P* for interaction = .636) (Table 2 and Central Illustration).

**Table 2:** Count of events (%) and HR estimates from Cox proportional hazard models on clinical outcomes for the REVIVED-BCIS2 trial and the STICH trial.

	REVIVED-BCIS2 trial				STICH trial			
	PCI, n (event rate per 100 patient-y)	OMT, n (event rate per 100 patient-y)	Unadjusted HR (95% CI)	*P* value for interaction	CABG, n (event rate per 100 patient-y)	OMT, n (event rate per 100 patient-y)	Unadjusted HR (95% CI)	*P* value for interaction
All-cause death								
Overall	105 (8.8)	105 (8.8)	1.00 (0.76-1.31)	–	345 (9.1)	385 (10.9)	0.84 (0.72-0.97)	–
EuroSCORE-II tertile 1	11 (3.8)	14 (4.2)	0.95 (0.43-2.1)	.793	98 (6.2)	125 (8.3)	0.75 (0.57-0.97)	.636
EuroSCORE-II tertile 2	28 (6.4)	34 (7.7)	0.83 (0.50-1.37)		115 (9.7)	127 (10.8)	0.89 (0.69-1.14)	
EuroSCORE-II tertile 3	66 (14.2)	57 (13.6)	1.03 (0.72-1.47)		132 (13.2)	133 (15.9)	0.84 (0.66-1.07)	
Hospitalization for HF								
Overall	51 (4.6)	50 (4.4)	1.03 (0.70-1.51)	–	152 (4.7)	199 (6.9)	0.71 (0.58-0.87)	–
EuroSCORE-II tertile 1	5 (1.8)	8 (2.5)	0.73 (0.24-2.20)	.802	44 (3.0)	64 (5.0)	0.66 (0.46-0.96)	.627
EuroSCORE-II tertile 2	13 (3.2)	15 (3.6)	0.92 (0.44-1.91)		43 (4.2)	66 (6.9)	0.63 (0.43-0.91)	
EuroSCORE-II tertile 3	33 (8.0)	27 (6.9)	1.07 (0.65-1.76)		65 (8.4)	69 (10.9)	0.80 (0.58-1.11)	
Cardiovascular death								
Overall	73 (6.1)	83 (6.9)	0.86 (0.63-1.18)	–	239 (6.3)	293 (8.2)	0.78 (0.66-0.91)	–
EuroSCORE-II tertile 1	8 (2.7)	9 (2.7)	1.08 (0.42-2.77)	.708	70 (4.4)	96 (6.3)	0.72 (0.53-0.97)	.851
EuroSCORE-II tertile 2	17 (3.9)	26 (5.9)	0.66 (0.37-1.19)		78 (6.5)	98 (8.1)	0.79 (0.59-1.05)	
EuroSCORE-II tertile 3	48 (10.4)	48 (11.5)	0.86 (0.58-1.26)		91 (9.0)	99 (11.8)	0.81 (0.62-1.06)	
All-cause death or HF hospitalization								
Overall	124 (11.2)	122 (10.8)	1.04 (0.81-1.33)	–	393 (12.1)	441 (15.4)	0.81 (0.70-0.93)	–
EuroSCORE-II tertile 1	15 (5.3)	19 (5.9)	0.93 (0.47-1.8)	.759	120 (8.3)	154 (11.9)	0.71 (0.56-0.90)	.548
EuroSCORE-II tertile 2	34 (8.4)	39 (9.4)	0.89 (0.56-1.4)		128 (12.5)	146 (15.4)	0.83 (0.65-1.05)	
EuroSCORE-II tertile 3	75 (18.1)	64 (16.4)	1.08 (0.77-1.50)		145 (18.6)	141 (22.5)	0.87 (0.69-1.09)	

CABG, coronary artery bypass grafting; EuroSCORE, European System for Cardiac Operative Risk Evaluation; HF, heart failure; HR, hazard ratio; OMT, optimal medical therapy; PCI, percutaneous coronary intervention; REVIVED-BCIS2, Revascularization for Ischemic Ventricular Dysfunction – British Cardiovascular Intervention Society 2; STICH, Surgical Treatment for Ischemic Heart Failure.

**Central Illustration:**
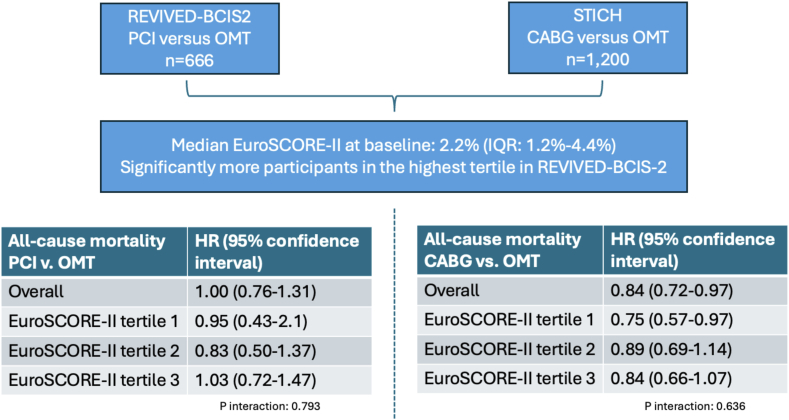
**All-cause mortality with revascularization in patients with ischemic left ventricular dysfunction according to baseline surgical risk.** CABG, coronary artery bypass grafting; EuroSCORE, European System for Cardiac Operative Risk Evaluation; HR, hazard ratio; OMT, optimal medical therapy; PCI, percutaneous coronary intervention; REVIVED-BCIS2, Revascularization for Ischemic Ventricular Dysfunction – British Cardiovascular Intervention Society 2; STICH, Surgical Treatment for Ischemic Heart Failure.

**Figure 2:**
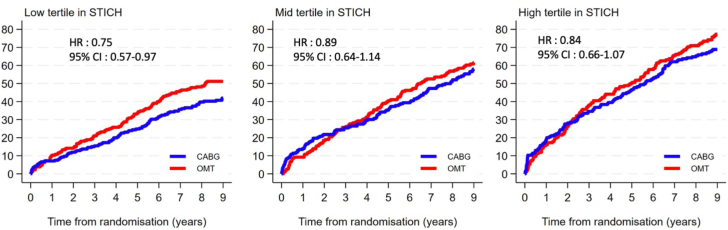
**All-cause mortality by tertiles of European System for Cardiac Operative Risk Evaluation (EuroSCORE)-II with coronary artery bypass grafting (CABG) or optimal medical therapy (OMT) in the Surgical Treatment for Ischemic Heart Failure (STICH) trial.** CABG reduced mortality consistently across tertiles of baseline EuroSCORE-II (*P* for interaction = .636). HR, hazard ratio.

**Figure 3:**
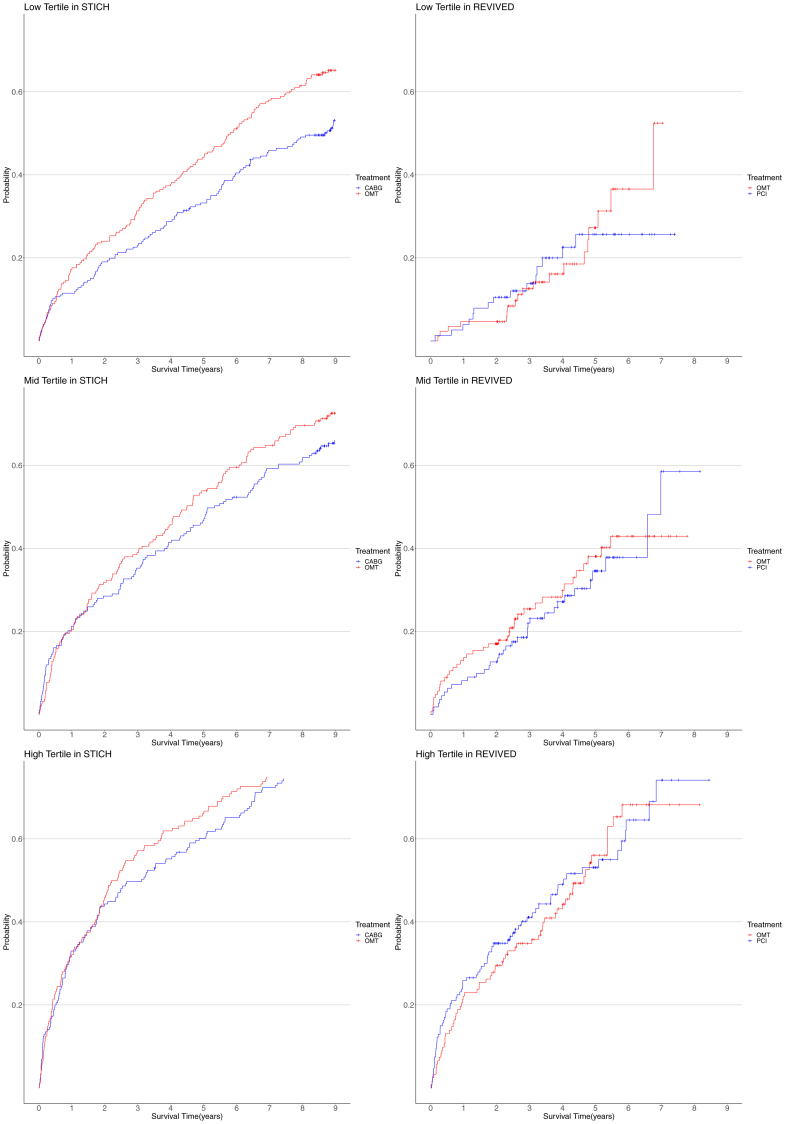
**Composite of all-cause mortality or heart failure (HF) hospitalization by tertiles of European System for Cardiac Operative Risk Evaluation (EuroSCORE)-II in Revascularization for Ischemic Ventricular Dysfunction – British Cardiovascular Intervention Society 2 (REVIVED-BCIS2) and Surgical Treatment for Ischemic Heart Failure (STICH) trials.** In the REVIVED-BCIS2 trial, percutaneous coronary intervention did not reduce the composite of all-cause mortality or HF hospitalization consistently across tertiles of baseline EuroSCORE-II (*P* for interaction = .759). In the STICH trial, coronary artery bypass grafting (CABG) reduced the composite of all-cause mortality or HF hospitalization consistently across tertiles of baseline EuroSCORE-II (*P* for interaction = .548). OMT, optimal medical therapy.

The cumulative incidence of all-cause mortality by CABG vs OMT in the STICH trial is shown by EuroSCORE-II tertile in Figure 2. The cumulative incidence of all-cause mortality at the 30-day landmark by tertiles is presented in Supplemental Figures S1-S3. In both trials, increasing EuroSCORE-II tertiles were associated with an increasing risk of all-cause mortality separately with PCI (*P* < .001), CABG (*P* < .001), and OMT (*P* < .001 in both REVIVED-BCIS2 and STICH trials). In the REVIVED-BCIS2 trial, the lack of treatment effect with PCI vs OMT alone on the rates of all the secondary outcomes was consistent across the spectrum of baseline EuroSCORE-II values (Table 2). In the STICH trial, the rates of all the secondary outcomes were lower with CABG vs OMT with no difference in treatment effect across baseline EuroSCORE-II values (Table 2). The cumulative incidence of the composite of all-cause death and HF hospitalization (REVIVED-BCIS2 primary end point) by tertiles is presented in Figure 3.

The results were consistent in a sensitivity analysis imputing different values to missing EuroSCORE-II variables to evaluate the impact of missingness in baseline variables (Supplemental Table S3). In the REVIVED-BCIS2 trial, restricted mean survival time was similar for PCI vs OMT across all EuroSCORE-II tertiles for the primary and all secondary outcomes (Supplemental Table S4). In the STICH trial, the restricted mean survival time was longer for the primary outcome and for all secondary outcomes in the lowest risk EuroSCORE-II tertile (Supplemental Table S5).

## Discussion

In this combined analysis of the 2 adequately powered RCT that have evaluated the impact of revascularization on cardiovascular outcomes in patients with ischemic LV systolic dysfunction, the main findings are that: (1) participants enrolled in the REVIVED-BCIS2 trial were older and had higher baseline surgical risk (as assessed by modified EuroSCORE-II) than patients enrolled in the STICH trial; (2) the treatment effect of PCI vs OMT alone in the REVIVED-BCIS2 trial did not differ across baseline surgical risk, and PCI was consistently not associated with a reduction in all-cause mortality; and (3) the treatment effect of CABG vs OMT alone in the STICH trial did not differ across baseline surgical risk, and CABG was consistently associated with a reduction in all-cause mortality. These observations held for the primary and secondary outcomes.

Following the publication of the REVIVED-BCIS2 trial, many hypotheses were raised to explain the apparent difference in treatment effect of PCI and CABG in this population, including the possibility that REVIVED-BCIS2 patients may have had a higher risk profile at randomization.^16^ In the REVIVED-BCIS2 trial, it is likely that participants who were deemed best served by CABG were referred for CABG and were not approached for the trial.^17^ This resulted in different patient populations in the REVIVED-BCIS2 (equipoise between PCI and OMT but felt not to be best served by CABG) and STICH (equipoise between CABG and OMT but not felt to be best served by PCI) trials. In this analysis, this hypothesis is reinforced by the observation that patients in the REVIVED-BCIS2 trial were almost 10 years older than those from the STICH trial, and that the baseline surgical risk was higher in the REVIVED-BCIS2 trial. Previous meta-analyses of observational studies and/or RCT subgroups, which suggested that CABG may be superior to PCI in patients with ischemic LV dysfunction, are likely to have included PCI patients who were not accepted for surgery due to perceived excessive surgical risk.^18^ The current analysis supports the fact that patients in the 2 trials represent 2 different clinical phenotypes that cannot be compared directly.

For the abovementioned reason, our objective was to evaluate the potential treatment-modifying effect of baseline surgical risk on the impact of revascularization on cardiovascular outcomes. We decided a priori to conduct the analyses separately within each trial data set to allow evaluation of the effect of revascularization modality on comparable patients treated with OMT alone. The neutral effect of PCI over OMT was consistent across the spectrum of baseline surgical risk, which is consistent with previous trials showing that PCI does not impact mortality in stable ischemic heart disease, many of which included only small numbers of patients with LV dysfunction.^19^, ^20^, ^21^ Because patients enrolled in the REVIVED-BCIS2 trial had a higher baseline risk, it is uncertain whether revascularization with PCI could improve clinical outcomes in a lower-risk population, similar to the STICH population. Baseline EuroSCORE-II was not an effect modifier in the STICH trial either, with a consistent reduction in all-cause mortality with CABG across the spectrum. The restricted mean survival time analysis (which considers potential early effects of the treatment, such as early peri-procedural complications) suggested that CABG was superior to OMT for the main outcome and all secondary outcomes in the lowest risk EuroSCORE-II tertile only. Whether the benefits of CABG observed in the STICH trial would still be observed if the study population had been similar to the REVIVED-BCIS2 population, with higher baseline surgical risk and according to current OMT practices, remains open for debate. Of note, overall baseline risk was low in the cohort, with a median EuroSCORE-II in the pooled data set of 2.2% with a narrow distribution. The small number of very high-risk patients in the data set limits our understanding of the potential effect modification of baseline risk on the treatment effect of revascularization across the spectrum of risk encountered in routine clinical practice. It is also possible that higher risk subgroups based on anatomy, and not on a surgical risk score, are more likely to show a benefit of either revascularization strategy.

Considering that PCI is used routinely in clinical practice in patients with HF, LV systolic dysfunction, and multivessel disease,^10^, ^11^, ^12^ despite guidelines recommendations favoring CABG, there remains a clinical equipoise among clinicians. This conundrum can only be addressed through a properly powered RCT comparing contemporary PCI vs CABG in a population of patients who are eligible for both revascularization strategies, avoiding the inevitable selection bias of previous observational studies regarding baseline risk in both groups. The STICH-3.0 International Trial Consortium (NCT05761067) includes independent RCT from Sweden, Denmark, Canada, and the United Kingdom, comparing PCI to CABG in this population.^9^^,^^22^ Other countries involved in the STICH-3.0 Consortium include the United States, Australia, New Zealand, the Netherlands, Germany, and France. Using harmonized eligibility criteria and key variables collected in the case report forms, this initiative will culminate in a pooled data set of individual patients that will yield the power to evaluate for the first time the comparative effectiveness of CABG vs PCI in terms of all-cause mortality in this population.

### Limitations

First, approximations were required to calculate the modified EuroSCORE-II, and some variables were unavailable, as detailed in Supplemental Table S1 (eg, chronic lung disease, active endocarditis, dialysis [in the STICH trial]), which may underestimate the baseline risk. It is, however, unlikely that patients in either trial had endocarditis or were on dialysis. The modified EuroSCORE-II in this study is also within the same range as the full EuroSCORE-II seen in routine clinical practice in this patient population, suggesting our study population is similar to real-world patients in that aspect.^23^ Due to randomization, it is expected that this potential bias would have impacted the comparator groups to the same extent within each trial. Second, baseline surgical risk was overall low, and the variability of its distribution was relatively small. Whether surgical risk could be an effect modifier in patients undergoign PCI in a higher risk cohort remains possible. Third, because of the differences in patient characteristics between the trials, the individual data were not pooled. Fourth, female participants were underrepresented (12% in both trials); therefore, the results may not be generalizable to all sexes. Finally, this post hoc analysis is exploratory and potentially underpowered, and future work should aim at assessing outcomes for CABG and PCI compared to OMT in ischemic cardiomyopathy as a function of baseline risk.

## Conclusion

In this combined analysis of REVIVED-BCIS2 and STICH, the 2 largest RCT evaluating the impact of revascularization strategies in patients with ischemic LV dysfunction and multivessel disease, the treatment effect of PCI vs OMT, and of CABG vs OMT, was not modified by baseline surgical risk.
