# Supplementary material for: Surgical Risk and Long-Term Mortality With PCI and CABG in Ischemic Left Ventricular Systolic Dysfunction
Source: J Soc Cardiovasc Angiogr Interv. 2025 Aug 19;4(9):103820. doi: 10.1016/j.jscai.2025.103820 (PMC12485539; doi:10.1016/j.jscai.2025.103820)
Supplement: Supplemental Tables and Figures [file mmc1.docx]

**Supplementary methods**

Due to missing data for components of the EuroSCORE-II, the sample sizes between the original trials and the current analysis differ. Specifically, EQ-5D mobility scores had 19 missing values, and creatinine clearance measurements had 13 missing values. These missing components accounted for 28 out of the 34 total missing values, resulting in 666 observations out of 700 (in REVIVED-BCIS2), and 1,200 observations out of 1,212 (in STICH) being used in the respective analyses.

**Supplemental Table S1. EuroSCORE-II variable definitions used in the REVIVED-**

**BCIS2 and STICH datasets.**

| **EuroSCORE-II variables** | **Calculation in STICH** | **Calculation in REVIVED-BCIS2** |
| --- | --- | --- |
| **No approximations used** |  |  |
| Age | Age | Age |
| Biological sex | Biological sex | Biological sex |
| Renal impairment based on creatinine clearance using the Cockcroft-Gault formula | Actual creatinine clearance based on the Cockcroft-Gault formula | Actual creatinine clearance based on the Cockcroft-Gault formula |
| Diabetes on insulin | Diabetes on insulin | Diabetes on insulin |
| CCS angina class 4 | CCS angina class 4 | CCS angina class 4 |
| LV function (based on LVEF categories) | LV function (based on LVEF categories) | LV function (based on LVEF categories) |
| NYHA class | NYHA class | NYHA class |
| Surgery on thoracic aorta | All 0 | All 0 |
| **Approximations** |  |  |
| Chronic lung disease | All 0 (not captured in the dataset) | All 0 (not captured in the dataset) |
| Extracardiac arteriopathy | Peripheral vascular disease or stroke | Peripheral vascular disease or cardiovascular accident |
| Poor mobility | Patient unable to walk | Patient with severe problems walking or unable to walk on EQ-5D-5L |
| Previous cardiac surgery | Previous mitral valve repair or replacement, or previous CABG (previous CABG was an exclusion criteria) | Previous CABG |
| Active endocarditis | All 0 (not captured in dataset)* | All 0 |
| Critical preoperative state | All 0** | All 0*** |
| Dialysis | All 0 (not captured in dataset) | Patient on dialysis |
| Recent MI within 90 days | Any previous myocardial infarction**** | All 0***** |
| Pulmonary hypertension | All 0 (missing for 80%) | All 0 (not captured in the dataset) |
| Urgency of operation**, ***, ****, ***** |  |  |
| Urgent | Hospitalization before index procedure within 30 days. | All 0 |
| Emergency | All 0 | All 0 |
| Salvage | All 0 | All 0 |
| Weight of operation |  |  |
| Non-CABG | All 0 | All 0 |
| 2 procedures | Patients randomized to CABG group of STICH who had a concomitant mitral valve surgery and one other procedure. Patients randomized to medical therapy were coded 0. | All 0 |
| 3 procedures | All 0 | All 0 |

* Aortic valvular heart disease clearly indicating the need for aortic valve repair or replacement was an exclusion criteria.

** Cardiogenic shock (within 72 hours of randomization), as defined by the need for intraaortic balloon support or the requirement for intravenous inotropic support were exclusion criteria.

*** Decompensated heart failure requiring inotropic support, invasive or non-invasive ventilation or IABP/left ventricular assist device (LVAD) therapy <72 hours prior to randomization, and sustained VT/VF or appropriate ICD discharges <72 hours prior to randomisation were exclusion criteria.

****Recent acute MI judged to be an important cause of left ventricular dysfunction was an exclusion criteria.

***** Myocardial infarction < 6 weeks previously was an exclusion criteria.

**Supplemental Table S2. Clinical endpoint definitions**

| **Outcome** | **STICH** | **REVIVED** |
| --- | --- | --- |
| Cardiovascular death | Death will be considered to be of cardiac etiology unless there is an obvious non-cardiac cause. Defined as sudden death, or death attributed to recurrent myocardial infarction, heart failure, a cardiovascular procedure, stroke, or other cardiovascular etiology   - Sudden death: Defined as death that occurred suddenly and unexpectedly in which the time of death is known. 14 Witnessed Death due to: • An identified arrhythmia (ECG or at least monitor recording, or monitor witnessed arrhythmia either by a medic or a paramedic). • Cardiac arrest or cardiovascular collapse in absence of premonitory heart failure or myocardial infarction or other modes of death. • Patients resuscitated from a sudden cardiac arrest who later die of the sequelae of the event or similar patients who die during an attempted resuscitation. Or • Death that occurred in which the time of death is unknown. In this case, the interval between the time the patient was last seen and the time the death became known will be recorded. - Fatal pump failure: Death occurring after new or worsening symptoms and/or signs of heart failure. Patients who are being treated for heart failure and who have a sudden death as the terminal event will be classified as having a pump failure related death. Classified as: 1. Heart failure with secondary arrhythmic death. 2. Heart failure without secondary arrhythmic death. - Fatal MI: Fatal myocardial infarction may be adjudicated in any one of the following three scenarios: • Death occurring after a documented myocardial infarction in which there is not conclusive evidence of another cause of death. Patients who are being treated for myocardial infarction and who have a sudden death as the terminal event related to the MI will be classified as having a myocardial infarction related death. • Autopsy evidence of a recent infarct with no other conclusive evidence of another cause of death. • A Fatal Myocardial Infarction may be adjudicated for an abrupt death that has suggestive criteria for an infarct but does not meet the strict definition of a myocardial infarction. The suggestive criteria is as follows: - Presentation of chest pain AND - One of the following: - ECG changes indicative of a myocardial injury or 15 - Abnormal markers without evolutional changes (i.e., patient died before a subsequent draw) or - other evidence of wall motion abnormality - Fatal CVA: Death occurring after a documented CVA. - Cardiovascular procedure related death: Death occurring during a cardiovascular procedure (CABG, SVR, PTCA, other) or when the events leading to death are related to the procedure. The type of procedure will be specified. (Example: A patient who had a CABG up to 15 days ago, who developed a subsequent myocardial infarction requiring inotropics, and who later died will still be classified as procedural related death.) - Surgical Ventricular Restoration Related Death: Death occurring after a surgical ventricular restoration where there is evidence the death was due primarily to the SAVR procedure versus CABG. - Other cardiovascular death: Death must be due to a fully documented cardiovascular cause not included above. | All deaths where there is no clinical or post-mortem evidence of a non-cardiovascular aetiology |
| Heart failure hospitalization | Congestive Heart Failure (CHF) will be adjudicated in the case of an unplanned presentation for new or worsening heart failure requiring an overnight stay in which the patient receives treatment with parenteral therapy including diuretics, inotropic, or vasodilator agents. In the absence of documentation of these therapies, description of significant diuresis will be considered as criteria for heart failure therapy. | Hospital admission (lasting >24 hours) for deteriorating symptoms or signs of heart failure, where there is a documented diagnosis of heart failure and the patient receives initiation or intensification of treatment for heart failure. Initiation or intensification of treatment includes at least one of the following:   - increase in oral diuretic dose or addition of another oral diuretic; - intravenous vasoactive therapy (vasodilator, inotrope or vasopressor); - mechanical circulatory support (MCS) (including intra-aortic balloon pump (IABP), Impella, extra-corporeal membrane oxygenation (ECMO)); or - cardiac transplantation.   Elective admission for implantation or revision of ICD/cardiac resynchronisation therapy (CRT) devices will NOT constitute an endpoint. |

**Supplemental Table S3. Interaction between EuroSCORE-II tertiles and randomized treatment for all-cause death in a sensitivity analysis imputing different values to missing EuroSCORE-II variables.**

|  | **Unadjusted HR* for PCI or CABG versus OMT (95% CI)** | **P-value for interaction** |
| --- | --- | --- |
| **REVIVED-BCIS2** |  |  |
| Overall | 0.98 (0.75-1.27) | 0.594 |
| EuroSCORE-II tertile 1 | 0.94 (0.44-1.99) |  |
| EuroSCORE-II tertile 2 | 0.80 (0.48-1.35) |  |
| EuroSCORE-II tertile 3 | 0.98 (0.69-1.39) |  |
| **STICH** |  |  |
| Overall | 0.84 (0.73-0.96) | 0.557 |
| EuroSCORE-II tertile 1 | 0.75 (0.57-0.97) |  |
| EuroSCORE-II tertile 2 | 0.90 (0.70-1.15) |  |
| EuroSCORE-II tertile 3 | 0.84 (0.66-1.06) |  |

* Hazard Ratios.

**Supplemental Table S4. Restricted mean survival times in days with PCI and OMT by tertiles of EuroSCORE-II in the REVIVED-BCIS2 trial.**

|  | **PCI** | **OMT*** | **p-value** |
| --- | --- | --- | --- |
| **Tertile 1** |  |  |  |
| All-cause death | 2259.4247 | 2221.5206 | 0.745 |
| Cardiovascular Death | 2339.2747 | 2346.7001 | 0.942 |
| Hospitalization for HF | 2419.8890 | 2365.1826 | 0.557 |
| All-cause Death or HF Hospitalization | 2147.0195 | 2104.6952 | 0.748 |
| **Tertile 2** |  |  |  |
| All-cause death | 2204.7393 | 2132.3490 | 0.596 |
| Cardiovascular Death | 2408.0238 | 2273.4861 | 0.308 |
| Hospitalization for HF | 2523.8969 | 2495.1474 | 0.805 |
| All-cause Death or HF Hospitalization | 2068.4318 | 2019.0076 | 0.741 |
| **Tertile 3** |  |  |  |
| All-cause Death | 1729.0274 | 1791.1970 | 0.678 |
| Cardiovascular Death | 1981.9545 | 1915.5315 | 0.673 |
| Hospitalization for HF | 2250.9520 | 2304.4140 | 0.734 |
| All-cause Death or HF Hospitalization | 1557.9776 | 1635.3146 | 0.614 |

* Optimal medical therapy.

**Supplemental Table S5. Restricted mean survival times in days with CABG and OMT by tertiles of EuroSCORE-II in the STICH trial.**

|  | **CABG*** | **OMT** | **p-value** |
| --- | --- | --- | --- |
| **Tertile 1** |  |  |  |
| All-cause death | **2729.3276** | **2480.2459** | **0.035** |
| Cardiovascular Death | **2923.8356** | **2653.3679** | **0.022** |
| Hospitalization for HF | **3207.6903** | **2980.0730** | **0.029** |
| All-cause Death or HF Hospitalization | **2434.1405** | **2096.0297** | **0.008** |
| **Tertile 2** |  |  |  |
| All-cause death | 2348.3592 | 2266.7630 | 0.546 |
| Cardiovascular Death | 2629.9475 | 2448.3230 | 0.191 |
| Hospitalization for HF | **3068.6632** | **2767.5897** | **0.019** |
| All-cause Death or HF Hospitalization | 2010.0149 | 1822.0032 | 0.183 |
| **Tertile 3** |  |  |  |
| All-cause Death | 1986.6156 | 1862.3336 | 0.377 |
| Cardiovascular Death | 2296.8555 | 2159.2558 | 0.364 |
| Hospitalization for HF | 2660.1189 | 2392.3810 | 0.094 |
| All-cause Death or HF Hospitalization | 1547.0977 | 1389.0185 | 0.271 |

* Coronary artery bypass graft.

**Supplemental Figure S1. Landmark analysis at 30 days of all-cause mortality with PCI and OMT (REVIVED-BCIS2) and CABG and OMT (STICH) in the first tertile of baseline EuroSCORE-II.**

**
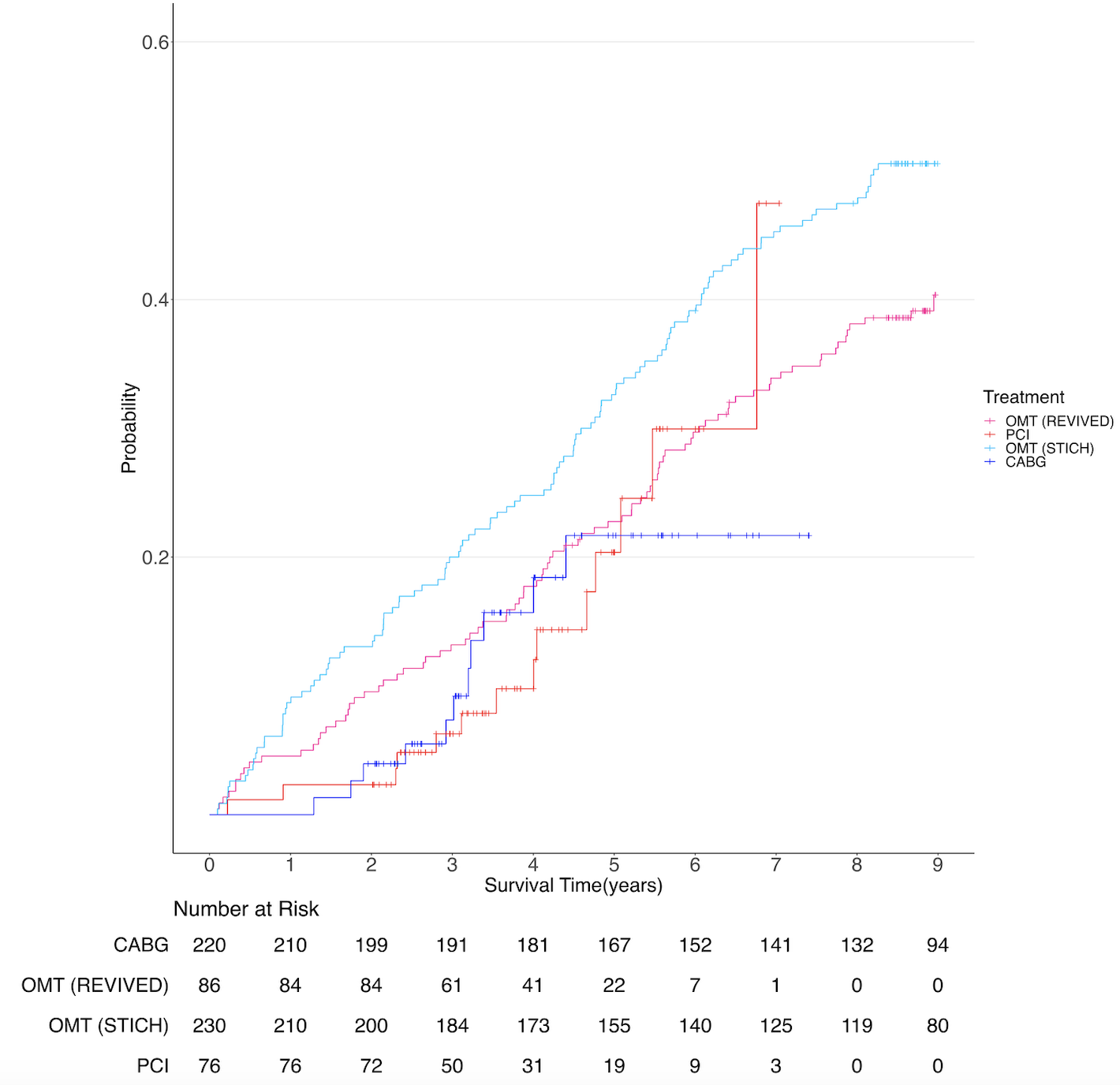
**

**Supplemental Figure S2. Landmark analysis at 30 days of all-cause mortality with PCI and OMT (REVIVED-BCIS2) and CABG and OMT (STICH) in the second tertile of baseline EuroSCORE-II.**

**
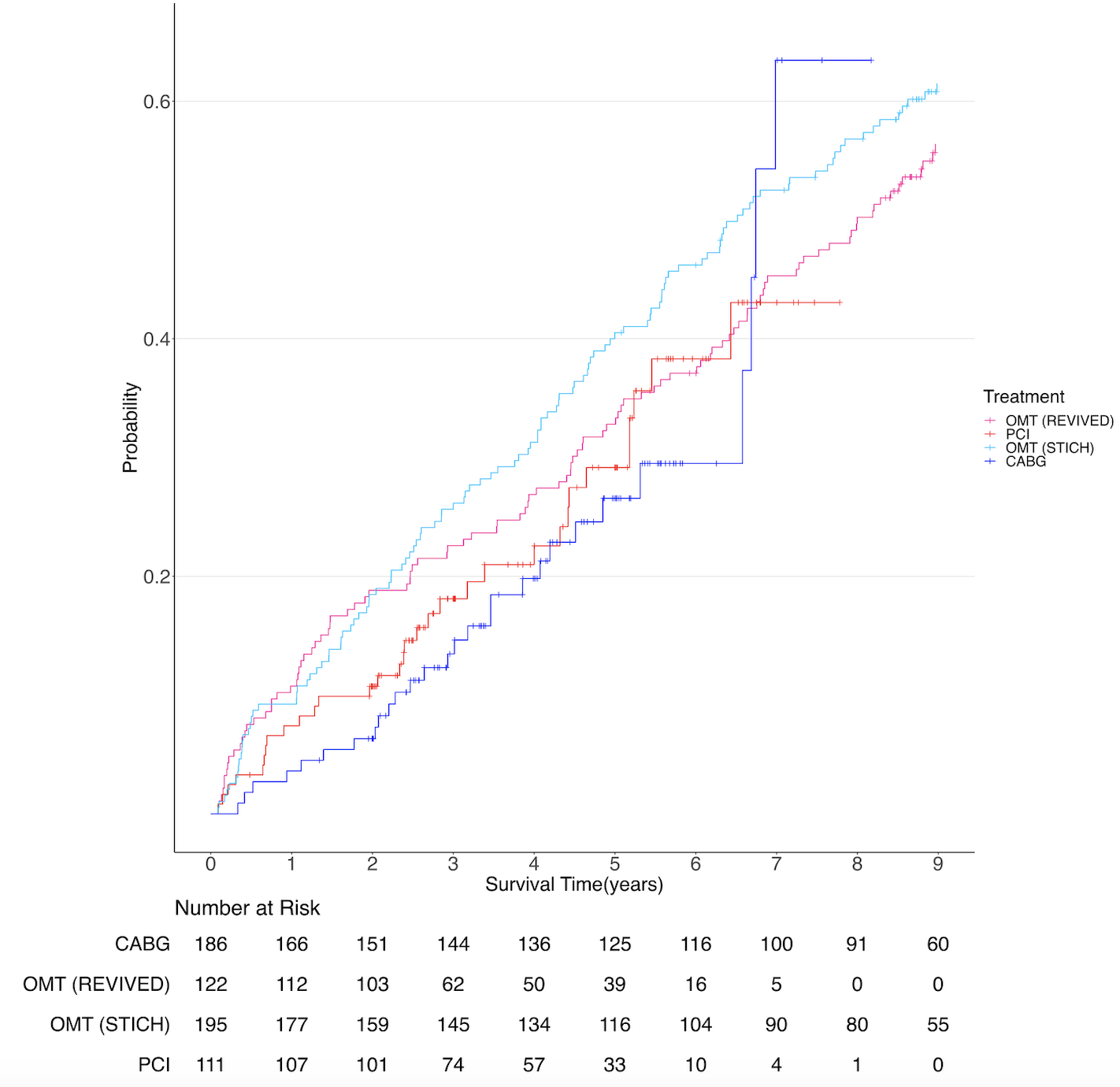
**

**Supplemental Figure S3. Landmark analysis at 30 days of all-cause mortality with PCI and OMT (REVIVED-BCIS2) and CABG and OMT (STICH) in the third tertile of baseline EuroSCORE-II.**

**
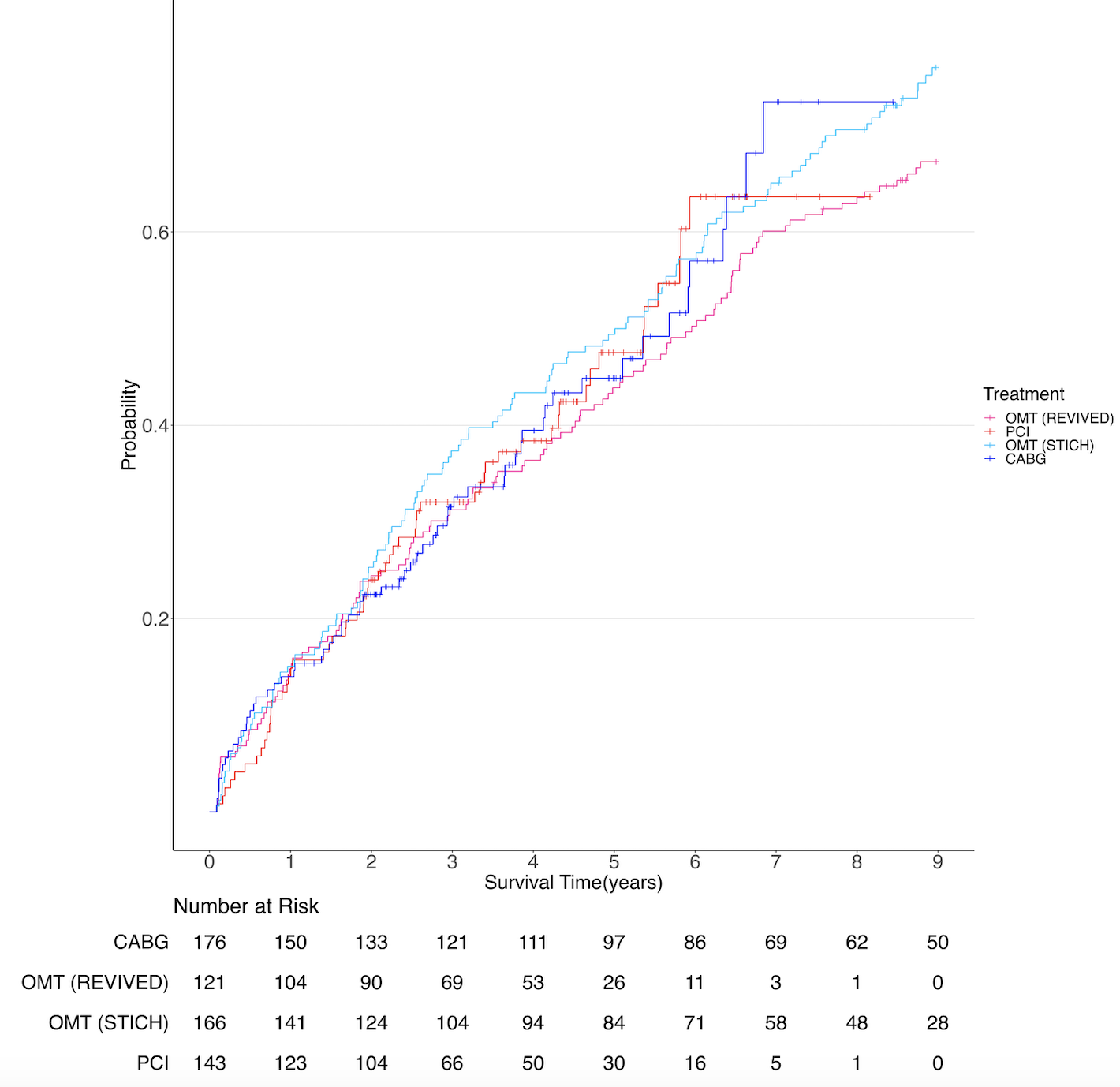
**
